# O.6.1-4 A whole school, systems-based approach to health enhancing physical activity (WSA HEPA)- challenges and lessons learnt from an ERASMUS+ Collaborative Partnership project

**DOI:** 10.1093/eurpub/ckad133.263

**Published:** 2023-09-11

**Authors:** Diane Crone, Paul Sellars, Vasillis Barkoukis, Timo Jaakkola, Miko Huhtiniemi

**Affiliations:** Cardiff Metropolitan University; Cardiff Metropolitan University; Aristotle University of Thessaloniki; University of Jyvaskyla; dmcrone@cardiffmet.ac.uk; University of Jyvaskyla; dmcrone@cardiffmet.ac.uk

## Abstract

**Purpose:**

Health-enhancing physical activity (HEPA) is an ongoing concern for all countries and in particular for young people, who are the future adults in our society. Global physical inactivity levels remain static (Guthold et al., 2018) and as a consequence there is an international goal to reduce physical inactivity by 10% by 2025 and by 15% by 2030 (WHO, 2018). To support the achievement of physical activity goals, the World Health Organisation designed a Global Action Plan for Physical Activity (GAPPA) (WHO, 2018). Using the GAPPA, the aim of the WSA HEPA project was to co-produce resource materials that promote the GAPPA objectives and adopt a whole-systems approach to addressing HEPA in schools for young people. In summary, this presentation will present the WSA HEPA project, explain the material development and how to access them (here) and conclude with challenges and lessons learnt.

**Description:**

The presentation will include (i) an overview of the project including the co-production approach to the material (module) development and EU wide mixed method needs analysis undertaken to inform their development; (ii) an explanation of the six modules and conclude with (iii) challenges and lessons learnt from the project.

**Conclusion:**

Participants attending the presentation will gain an understanding of how a whole school, whole systems-based approach for health enhancing physical activity can be implemented. They will also have access to the resources to enable this from the project and be cognisant of the challenges and benefits to such an approach. They should have sufficient knowledge and access to resources to be able to implement this in their home countries.

